# RNA-Seq analysis of the fruiting bodies and mycelia of angel-wing mushroom *Pleurocybella porrigens* that cause acute encephalopathy

**DOI:** 10.1186/s13104-024-06860-2

**Published:** 2024-07-24

**Authors:** Nozomu Watanabe, Keisuke Mitsukuni, Takumi Sato, Jili Zhang, Akiko Ono, Tomohiro Suzuki

**Affiliations:** 1 Center for Bioscience Research and Education, Utsunomiya University, Tochigi, 321-8505 Japan; 2 Present Address: Faculty of Global Interdisciplinary Science and Innovation, Shizuoka University, 836 Ohya, Suruga-ku, Shizuoka, 422-8529 Japan

**Keywords:** Fungi, Gene expression analysis, Lectin, Real-time RT-PCR, Transcriptome

## Abstract

**Objective:**

In 2004, after consuming angel-wing mushrooms, *Pleurocybella porrigens*, 59 incidents of food poisoning were reported in Japan. Consequently, 17 individuals died of acute encephalopathy. In 2023, we proved that a lectin, pleurocybelline, and pleurocybellaziridine from this mushroom caused damage to the brains of mice. Although we reported genomic and transcriptomic data of *P. porrigens* in 2013, the assembly quality of the transcriptomic data was inadequate for accurate functional annotation. Thus, we obtained detailed transcriptomic data on the fruiting bodies and mycelia of this mushroom using Illumina NovaSeq 6000.

**Results:**

*De novo* assembly data indicated that the N50 lengths for the fruiting bodies and mycelia were improved compared with those previously reported. The differential expression analysis between the fruiting bodies and the mycelia revealed that 1,937 and 1,555 genes were significantly up-regulated in the fruiting bodies and the mycelia, respectively. The biological functions of *P. porrigens* transcripts, including PA biosynthetic pathways, were investigated using BLAST search, Gene Ontology, and Kyoto Encyclopedia of Genes and Genomes pathway analysis. The obtained results revealed L-valine, a predicted precursor of PA, is biosynthesized in the fruiting bodies and mycelia. Furthermore, real-time RT-PCR was performed to evaluate the accuracy of the results of differential expression analysis.

**Supplementary Information:**

The online version contains supplementary material available at 10.1186/s13104-024-06860-2.

## Introduction

The mushroom *Pleurocybella porrigens* (Japanese name, Sugihiratake; English name, Angel-wing mushroom) belongs to the family Tricholomataceae. It is widely distributed in temperate regions of the Northern Hemisphere, where it grows in overlapping clumps on old stumps and fallen trees of conifers such as cedar and pine [1, 2]. One characteristic feature of the fruiting bodies is that they grow in clusters of overlapping ear- or fan-shaped white umbrellas ranging between 2 and 6 cm in diameter. *P. porrigens* has been consumed worldwide, particularly in Asia. However, in autumn 2004, 59 incidents of food poisoning were reported in Japan. Of them, 17 individuals died from acute encephalopathy [3–5]. Many of them had chronic renal failure and underwent hemodialysis prior to death. As a result, the Ministry of Health, Labour and Welfare of Japan launched a study group to elucidate food poisoning caused by *P. porrigens*; however, the group was disbanded in 2006 after concluding that the disease was of unexplained origin. To date, vitamin D analogs [6], fatty acids [7], saccharides [8], and hydrogen cyanide [9] have been reported as potential causative toxins as components of *P. porrigens*.

We have previously reported some research related to food poisoning caused by *P. porrigens* [10–13]. In 2023, we demonstrated that a mixture of pleurocybelline (PC) and *P. porrigens* lectin (PPL) exhibited exo-protease activity, degrading various substrates at the *N-* and *C-*termini, with no amino acid specificity. Furthermore, when the mixture of PC, PPL, and pleurocybellaziridine (PA) was administered to mice, the number of apoptotic cells significantly increased in the hippocampus. Thus, we concluded that the PPL-PC complex damages the blood-brain barrier (BBB). Subsequently, the PA causes acute encephalopathy [14, 15] (Additional file 1: Figure S1). PA is a novel compound containing an aziridine skeleton at that time. Although Wakimoto et al. carried out a total synthesis study and demonstrated its existence in nature, its biosynthetic pathway remains unclear [16]. In addition, in 2013, we reported the genomic and transcriptomic data of the fruiting bodies and mycelia of *P. porrigens* using the Illumina Genome Analyzer (GAIIx) [17]. However, the transcriptomic data obtained comprised short reads (100 bp in length). Moreover, the N50 lengths, a measure of assembly quality, were 1,069 bp for the fruiting bodies and 633 bp for mycelia [17]; they were of lower quality than that of the current technology. Furthermore, there was an inevitable divergence in the expression differences between the Reads Per Kilobase of exon per Million mapped reads (RPKM), a computational analysis, and a semi-quantitative RT-PCR analysis. Previous genomic and transcriptomic data for the fruiting bodies and mycelia have been reported in the integrated genome database A-WING [18].

The objectives of this study were to obtain highly accurate transcriptomic data on the fruiting bodies and mycelia of *P. porrigens* using a next-generation sequencer (Illumina NovaSeq 6000) and to elucidate the biological functions of *P. porrigens*, including the PA biosynthetic pathway, using a BLAST search, Gene Ontology (GO) analysis, and Kyoto Encyclopedia of Genes and Genomes (KEGG) pathway analysis.

## Materials and methods

mRNA extraction methods for the fruiting bodies and mycelia are described in Additional file 1: Material S1.

## Results and discussion

### Sequencing of mRNA

Short-read sequences (151 bp in length) of mRNA from *P. porrigens* were generated using Illumina NovaSeq 6000 (Additional file 1: Table S1). Subsequently, paired-end (PE) reads from mRNAs were generated: 155,071,264 reads (77,535,632 pairs) and 163,716,256 reads (81,858,128 pairs) from the fruiting bodies and mycelia, respectively. Trimmomatic ver. 0.39 [19] was used to obtain high-quality read sequences by removing adapter sequences, reads less than 75 bp, and regions with low-quality scores in FASTQ files (quality scores < 15), resulting in 136,821,024 (88.2%) and 140,981,096 high quality reads (86.1%) of the fruiting bodies and mycelia, respectively.

### *De novo* assembly

Based on the high-quality short reads obtained from RNA-Seq, unigenes (here, we refer to contigs obtained from transcriptomic sequences as unigenes) were created by *de novo* assembly using Trinity ver. 2.8.5 [20]. In this step, the Jaccard-clip option was selected to avoid the fusion of adjacent transcripts. The *de novo* assembly resulted in 17,747 (168,237,925 bp) and 12,330 (108,597,207 bp) unigenes in the fruiting bodies and mycelia, respectively. The N50 lengths were 3,384 bp for the fruiting bodies and 3,154 bp for the mycelia, with average lengths of 2,197 bp and 2,222 bp, respectively (Table 1). The total sizes of unigenes were 5–10 times larger, and the N50 lengths were 3–5 times larger than those reported in our previous study [17]. The quality of the assembly data obtained in this study was significantly higher than that reported previously [17].

**Table 1:** Assembly and annotation summary compared to the previous study in 2013

	Illumina Genome Analyzer (2013)		Illumina NovaSeq 6000 (This study)	
	Fruiting bodies	Mycelia	Fruiting bodies	Mycelia
Number of unigenes	45,390	26,216	17,747	12,330
Total size of unigenes (bp)	29,504,308	11,748,163	168,237,925	108,597,207
N50 (bp)	1069	633	3384	3154
Average length (bp)	650	448	2197	2222
Maximum length (bp)	9955	8825	17,164	17,310
Minimum length (bp)	100	100	179	201
Unigenes having significantly similar sequences in the nr database	2219	2834	3075	2865
Unigenes assigned to GO terms	11,101	5570	10,904	8850
Unigenes assigned to KO IDs	9085	5251	3619	3497

### Functional annotations for *P. porrigens* unigenes

**Fig. 1:**
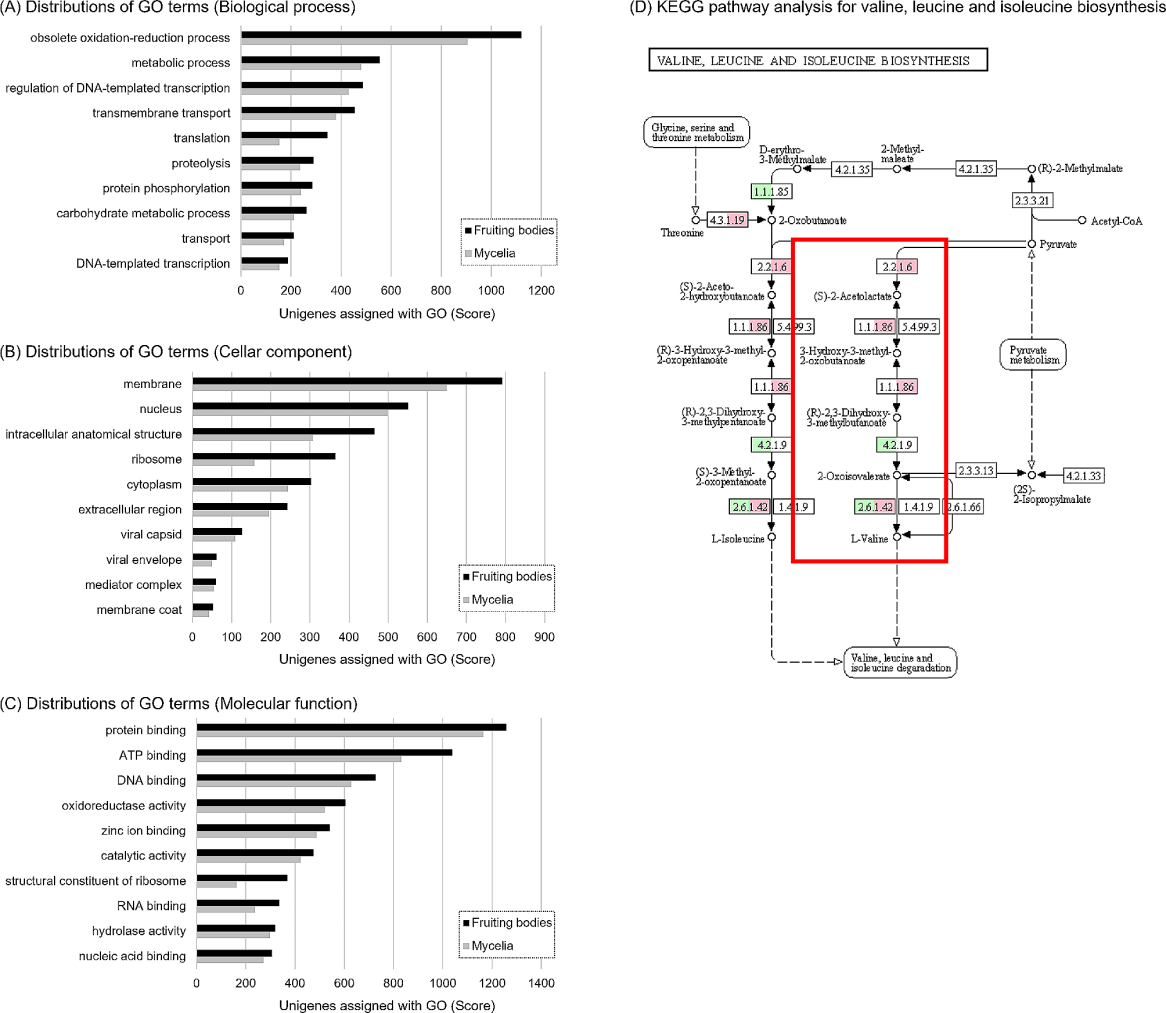
Result of Gene Ontology and KEGG pathway analysis. (**A**) Distributions of GO terms assigned to the unigenes to Biological process, (**B**) Cellular component, (**C**) Molecular function. (**D**) KEGG pathway analysis for valine, leucine and isoleucine biosynthesis. Green cells: Gene expression increased in the fruiting bodies. Pink cells: Gene expression increased in the mycelia

To predict the biological function of *P. porrigens* transcripts, we compared the unigenes in the fruiting bodies and mycelia with the NCBI non-redundant (nr) database using BLASTX [21] (Table 1). We observed significant homology with the nr database for 3,075 unigenes (17.3%) in the fruiting bodies and 2,865 unigenes (23.2%) in the mycelia. In addition, one or more GO terms [22] were assigned to 10,904 unigenes (61.4%) in the fruiting bodies and 8,850 unigenes (71.8%) in the mycelia. The distribution of GO categories (biological process, cellar component, molecular function) for unigenes assigned to GO terms was almost same (Additional file 1: Figure S2). In the biological process category, “obsolete oxidation-reduction process (GO:0055114)” and “translation (GO: 0006412)” were more frequently expressed in the fruiting bodies, while in the cellular component category, “intracellular anatomical structure (GO: 0005622)” and “ribosome (GO: 0005840)” were more frequently expressed in the fruiting bodies. Furthermore, in the molecular function category, “ATP binding (GO: 0005524)” and “structural constituent of ribosome (GO:0003735)” were more frequently expressed in the fruiting bodies (Fig. 1a-c). We found that “translation” in the biological process category, “ribosome” in the cellular component category, and “structural constituent of ribosome” in the molecular function category were expressed more in the fruiting bodies, implying that genes associated with transcription of DNA to mRNA and protein synthesis by translation of mRNA are activated during fruiting body formation. These results were consistent with the transcriptomic data of *P. porrigens* obtained in the previous study using GAIIx as a next-generation sequencer [17].

In the previous study, the N50 lengths were 1,069 and 633 bp in the fruiting bodies and mycelia, respectively [17]. These lengths are considered low compared to the current technology. Furthermore, discrepancies were observed between expression differences, as analyzed by RPKM [23] and semi-quantitative RT-PCR analysis. The results of RPKM, a computational analysis, left room for improvement.

Next, the differential expression analysis between the fruiting bodies and the mycelia was performed in this study. As a result, we found that 1,937 and 1,555 genes were significantly up-regulated in the fruiting bodies and mycelia, respectively (LogFC>|1|, FDR < 0.05) through differential expression analysis using RSEM [24], a software to quantify the number of transcripts from RNA sequence data. In addition, GO enrichment analysis revealed that “RNA-dependent RNA polymerase activity (GO: 0003968)”, “RNA processing (GO: 0006396)”, “sporulation (GO: 0043934)”, “mRNA methyltransferase activity (GO: 0008174)”, and “mRNA methylation (GO: 0080009)” were significantly expressed in the fruiting bodies (Additional file 1: Table S2). The significant expression of “RNA-dependent RNA polymerase activity” and “mRNA methyltransferase activity” in the fruiting bodies suggests that DNA to mRNA transcription and mRNA maturation are activated during fruiting body formation. These results are consistent with the functional annotations shown in Fig. 1. Furthermore, the significant expression of “sporulation” was consistent with the fact that the fruiting bodies form sporulate to produce offspring. Detailed data of the differential expression analysis are described in Additional file 2: Material S2.

### Metabolic pathway analysis for unigenes

KEGG [25, 26] pathway analysis was performed on 3,684 and 3,496 unigenes in the fruiting bodies and mycelia, respectively. As a result, 563 unigenes in the fruiting bodies and 614 unigenes in the mycelia were annotated as “Metabolic pathways (map01100)” (Additional file 1: Figure S3).

To analyze the biosynthetic pathway of PA, which was revealed to cause brain damage in the previous study, different gene expression levels in the biosynthetic pathway of L-valine, a predicted precursor, were confirmed (Fig. 1d) [14]. This is the first attempt to identify the biosynthetic pathway of PA by KEGG pathway analysis. KEGG pathway analysis showed that several unigenes in the fruiting body and mycelia were involved in the biosynthetic pathway of L-valine from pyruvate. In addition, some dehydrogenase activities, including “NADH dehydrogenase (ubiquinone) activity (GO:0008137)”, were more frequently expressed in the fruiting bodies, suggesting that the γ-position dehydrogenation is the presumed reaction mechanism for the biosynthesis of PA from L-valine (Additional file 1: Figure S4). These results are consistent with our preliminary data that while the leading cause of food poisoning by *P. porrigens* is from PA found in the fruiting bodies, it is also present in mycelia.

### Experimental analysis for the fruiting bodies and mycelia

**Fig. 2:**
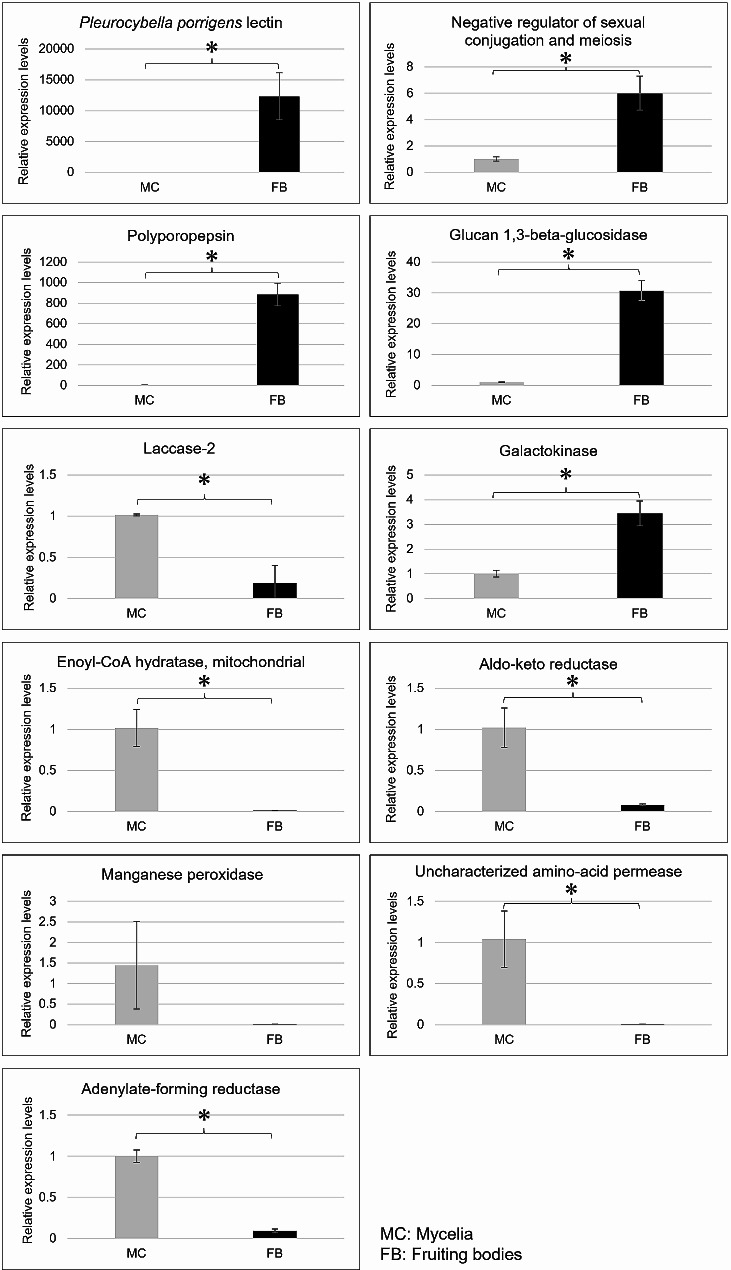
Differential expression analysis based on real-time RT-PCR validation results. Expression levels in the mycelia (MC) and the fruiting bodies (FB) are shown in gray and black, respectively

To evaluate the accuracy of the results of differential expression analysis, real-time RT-PCR was performed on 11 differentially expressed genes that showed significant homology from the result of BLAST search. “*Negative regulator of sexual conjugation and meiosis*”, “*polyporopepsin*”, “*glucan 1*,*3-beta-glucosidase*”, “*laccase-2*”, and “*galactokinase*”, the most upregulated genes in the fruiting bodies by RSEM, were selected as target genes for real-time RT-PCR. Five genes were selected as the most upregulated in the mycelia: “*enoyl-CoA hydratase*, mitochondrial”, “*aldo-keto reductase*”, “*manganese peroxidase*”, “*uncharacterized amino-acid permease*”, and “*adenylate-forming reductase*”. In addition, *ppl*, a lectin gene characteristic of *P. porrigens*, was also selected. Primers for real-time RT-PCR were designed to have a length of 20–25 bp and Tm values of 63–68 °C (Additional file 1: Table S3). The actin gene was used as an internal control. Real-time RT-PCR revealed that five of the six genes predicted to be significantly expressed in the fruiting bodies by RSEM showed high expression levels in the fruiting bodies. Four of the five genes predicted to be significantly expressed in the mycelia showed high expression levels in the mycelia (Fig. 2). Although *laccase-2* was predicted to be significantly expressed in the fruiting bodies, it showed high expression levels in the mycelia by real-time RT-PCR. *Manganese peroxidase* was predicted to be significantly expressed in the mycelia; however, there was a slight increase in expression in mycelia, and found to be not significant by real-time RT-PCR. Both laccase-2 and manganese peroxidase are oxidases secreted outside the fungus and may be susceptible to environmental factors [27, 28]. Nevertheless, most gene expressions were consistent with the expected results, indicating that the accuracy of differential expression analysis by RSEM in this study was higher than in previous studies. These results implied that more accurate transcriptomic data were obtained in this study.

## Conclusion

We conducted a transcriptome analysis using a next-generation sequencer (Illumina NovaSeq 6000) to re-acquire and improve the accuracy of transcriptomic data on the fruiting bodies and mycelia of *P. porrigens*. Comparison with the nr database using the BLAST search and GO enrichment analysis suggested that genes related to DNA transcription and translation are activated during fruiting body formation. The results of KEGG pathway analysis indicated that L-valine, a predicted precursor of PA, is likely biosynthesized both in the fruiting bodies and mycelia. In addition, the results of real-time RT-PCR and RSEM were identical for 9 out of 11 genes. This study demonstrates the improved performance of next-generation sequencers and provides new accurate and extensive transcriptomic data on *P. porrigens*, including differential expression level of PPL in the fruiting bodies and mycelia, as well as the suggested biosynthetic pathway of PA for future studies.

### Limitations

Identification of the enzymes involved in PA biosynthesis and mapping the acquired RNA-Seq to the genome sequence of *P. porrigens* were not included in this study.

### Electronic supplementary material

Below is the link to the electronic supplementary material.

Supplementary Material 1

Supplementary Material 2

## Data Availability

Sequencing data is available in DNA Data Bank of Japan (DDBJ) under accession number DRA017296.

## Abbreviations

BBB: Blood-brain barrier
FDR: False discovery rate
GO: Gene Ontology: KEGG, Kyoto Encyclopedia of Genes and Genomes
PA: Pleurocybellaziridine
PC: Pleurocybelline
PPL: Pleurocybella porrigens lectin
RPKM: Reads Per Kilobase of exon per Million mapped reads

## References

[1] Matsumoto T, Nagasawa E, Fukumasa-Nakai Y. Variation of ITS sequences in a natural Japanese population of *Pleurocybella porrigens*. Mycoscience. 2005;46(6):370–5. 10.1007/S10267-005-0262-8

[2] Anisworth GC. Ainsworth & Bisby’s dictionary of the fungi. CABI; 2008.

[3] Kuwabara T, Arai A, Honma N, Nishizawa M. Acute encephalopathy among patients with renal dysfunction after ingestion of sugihiratake, angel’s wing mushroom–study on the incipient cases in the northern area of Niigata Prefecture. Clin Neurol. 2005;45(3):239–45. 15835296

[4] Obara K, Okawa S, Kobayashi M, Takahashi S, Watanabe S, Toyoshima I. A case of encephalitis-type encephalopathy related to *Pleurocybella porrigens* (Sugihiratake). Clin Neurol. 2005;45(3):253–6. 15835299

[5] Obara K, Wada C, Yoshioka T, Enomoto K, Yagishita S, Toyoshima I. Acute encephalopathy associated with ingestion of a mushroom, *Pleurocybella porrigens* (angel’s wing), in a patient with chronic renal failure. Neuropathology. 2008;28(2):151–6. 18366348 10.1111/j.1440-1789.2007.00819.x

[6] Sasaki H, Akiyama H, Yoshida Y, Kondo K, Amakura Y, Kasahara Y, Tamio Maitani T. Sugihiratake Mushroom (Angel’s Wing Mushroom)-Induced Cryptogenic Encephalopathy may involve Vitamin D Analogues. Biol Pharm Bull. 2006;29(12):2514–8. 17142993 10.1248/bpb.29.2514

[7] Hasegawa T, Ishibashi M, Takata T, Takano F, Ohta T. Cytotoxic fatty acid from *Pleurocybella porrigens*. Chem PharmBull. 2007;55(12):1748–9. 10.1248/cpb.55.1748 18057752

[8] Takata T, Hasegawa T, Tatsuno T, Date J, Ishigaki I, Nakamura Y, Tomosugi N, Takano F, Ohta T. Isolation of N-acetylneuraminic acid and N-glycolylneuraminic acid from *Pleurocybella porrigens*. J Health Sci. 2009;55(3):373–9. 10.1248/jhs.55.373

[9] Gonmori K, Yokoyama K. Acute encephalopathy caused by cyanogenic fungi in 2004, and magic mushroom regulation in Japan. Chudoku Kenkyu. 2009;22(1):61–9. 19344063

[10] Suzuki T, Amano Y, Fujita M, Kobayashi Y, Dohra H, Hirai H, Murata T, Usui T, Morita T, Kawagishi H. Purification, characterization, and cDNA cloning of a lectin from the mushroom *pleurocybella porrigens*. Biosci Biotechnol Biochem. 2009;73(3):702–9. 19270381 10.1271/bbb.80774

[11] Kawaguchi T, Suzuki T, Kobayashi Y, Kodani S, Hirai H, Nagai K, Kawagishi H. Unusual amino acid derivatives from the mushroom *pleurocybella porrigens*. Tetrahedron. 2010;66(2):504–7. 10.1016/j.tet.2009.11.041

[12] Suzuki T, Dohra H, Omae S, Takeshima Y, Choi JH, Hirai H, Kawagishi H. Heterologous expression of a lectin from *Pleurocybella porrigens* (PPL) in *Phanerochaete Sordida* YK-624. J Microbiol Methods. 2014;100:70–6. 24631556 10.1016/j.mimet.2014.02.016

[13] Suzuki T, Nakamura L, Inayoshi S, Tezuka Y, Ono A, Choi JH, Dohra H, Sasanami T, Hirai H, Kawagishi H. An efficient heterologous Escherichia coli-based expression system for lectin production from *Pleurocybella porrigens*. Biosci Biotechnol Biochem. 2021;85(3):630–3. 33624769 10.1093/bbb/zbaa058

[14] Suzuki T, Asakawa T, Maekawa F, Kimura E, Tezuka Y, Nakamura L, Sato T, Arai Y, Choi JH, Suzuki M, et al. Possible molecular mechanism for acute encephalopathy by angel-wing mushroom ingestion - involvement of three constituents in onset-. Toxicon. 2023;221:106958. 36377137 10.1016/j.toxicon.2022.106958

[15] Kawagishi H. Chemical elucidation of acute encephalopathy by ingestion of angel-wing mushroom (*Pleurocybella porrigens*) - involvement of three constituents in onset. PJA Ser B. 2023;99(7):191–7. 10.2183/pjab.99.012 PMC10700016 37518008

[16] Wakimoto T, Asakawa T, Akahoshi S, Suzuki T, Nagai K, Kawagishi H, Kan T. Proof of the existence of an unstable amino acid: pleurocybellaziridine in *Pleurocybella porrigens*. Angew Chem Int Edit. 2011;50(5):1168–70. 10.1002/anie.201004646 21268219

[17] Suzuki T, Igarashi K, Dohra H, Someya T, Takano T, Harada K, Omae S, Hirai H, Yano K, Kawagishi H. A new omics data resource of *Pleurocybella porrigens* for gene discovery. PLoS ONE. 2013;8(7):e69681. 23936076 10.1371/journal.pone.0069681 PMC3720577

[18] Yamamoto N, Suzuki T, Kobayashi M, Dohra H, Sasaki Y, Hirai H, Yokoyama K, Kawagishi H, Yano K. A-WINGS: an integrated genome database for *Pleurocybella porrigens* (Angel’s wing oyster mushroom, sugihiratake). BMC Res Notes. 2014;7:866. 25465051 10.1186/1756-0500-7-866 PMC4289373

[19] Bolger AM, Lohse M, Usadel B. Trimmomatic: a flexible trimmer for Illumina sequence data. Bioinformatics. 2014;30(15):2114–20. 24695404 10.1093/bioinformatics/btu170 PMC4103590

[20] Grabherr MG, Haas BJ, Moran Y, Joshua ZL, Thompson DA, Amit I, Adiconis X, Fan L, Raychowdhury R, Zeng Q, et al. Trinity: reconstructing a full-length transcriptome without a genome from RNA-Seq data. Nat Biotechnol. 2011;29(7):644–52. 21572440 10.1038/nbt.1883 PMC3571712

[21] Altschul SF, Gish W, Miller W, Myers EW, Lipman DJ. Basic local alignment search tool. J Mol Biol. 1990;215(3):403–10. 2231712 10.1016/S0022-2836(05)80360-2

[22] Ashburner M, Ball CA, Blake JA, Botstein D, Butler H, Cherry JM, Davis AP, Dolinski K, Dwight SS, Eppig JT, et al. Gene ontology: tool for the unification of biology. The Gene Ontology Consortium. Nat Genet. 2000;25(1):25–9. 10802651 10.1038/75556 PMC3037419

[23] Mortazavi A, Williams BA, McCue K, Schaeffer L, Wold B. Mapping and quantifying mammalian transcriptomes by RNA-Seq. Nat Methods. 2008;5:621–8. 18516045 10.1038/nmeth.1226 PMC13303166

[24] Li B, Dewey CN. RSEM: accurate transcript quantification from RNA-Seq data with or without a reference genome. BMC Bioinf. 2011;12:323. 10.1186/1471-2105-12-323 PMC3163565 21816040

[25] Moriya Y, Itoh M, Okuda S, Yoshizawa AC, Kanehisa M. KAAS: an automatic genome annotation and pathway reconstruction server. Nucleic Acids Res. 2007;35(Web Server issue):182–5. 10.1093/nar/gkm321 PMC1933193 17526522

[26] Mao X, Cai T, Olyarchuk JG, Wei L. Automated genome annotation and pathway identification using the KEGG Orthology (KO) as a controlled vocabulary. Bioinformatics. 2005;21(19):3787–93. 15817693 10.1093/bioinformatics/bti430

[27] Zhong X, Li M, Zhang M, Feng Y, Zhang H, Tian H. Genome-wide analysis of the laccase gene family in wheat and relationship with arbuscular mycorrhizal colonization. Planta. 2022;257(1):15. 36528718 10.1007/s00425-022-04048-1

[28] Salame TM, Knop D, Levinson D, Mabjeesh SJ, Yarden O, Hadar Y. Release of Pleurotus Ostreatus versatile-peroxidase from Mn^2+^ repression enhances anthropogenic and natural substrate degradation. PLoS ONE. 2012;7(12):e52446. 23285046 10.1371/journal.pone.0052446 PMC3528650

