# Supplementary material for: RNA-Seq analysis of the fruiting bodies and mycelia of angel-wing mushroom Pleurocybella porrigens that cause acute encephalopathy
Source: BMC Res Notes. 2024 Jul 24;17:204. doi: 10.1186/s13104-024-06860-2 (PMC11270884; doi:10.1186/s13104-024-06860-2)
Supplement: Supplementary file 1 — Supplementary Material 1 [file 13104_2024_6860_MOESM1_ESM.docx]

Supplementary Information

**RNA-Seq analysis of fruiting bodies and the mycelia of angel-wing mushroom *Pleurocybella porrigens* that cause acute encephalopathy**

Nozomu Watanabe^a,1^, Keisuke Mitsukuni^a,1^, Takumi Sato^a^, JiLi Zhang^a^,

Akiko Ono^b^, Tomohiro Suzuki^a,*^

Correspondence to: suzukit@cc.utsunomiya-u.ac.jp

^a^ Center for Bioscience Research and Education, Utsunomiya University, Tochigi, 321-8505, Japan

^b^ Forestry and Forest Products Research Institute, 1 Matsunosato, Tsukuba, Ibaraki, 305-8687 Japan

^1^ These authors contributed equally to this work.

**Contents: Description**

**Table S1** The number of sequencing reads.

**Table S2** GO terms that are significantly expressed in fruiting bodies (Score≦5, FDR<0.05).

**Table S3** Proteins, primers, and product sizes for real-time RT-PCR.

**Figure S1** Proposed molecular mechanism of acute encephalopathy following ingestion of *Pleurocybella porrigens*.

**Figure S2** Distributions of GO categories assigned to the unigenes.

**Figure S3** Distributions of KEGG assigned to the unigenes.

**Figure S4** Dehydrogenase-related unigenes assigned to GO term (Score).

**Material S1** Materials and methods: Sample preparation for RNA-seq.

**Material S2** Detailed data from the differential expression analysis of *Pleurocybella porrigens*.

Table S1

The number of sequencing reads.

| Library | Number of raw reads | Number of  high-quality reads |
| --- | --- | --- |
| Fruiting bodies (PE reads, n=3) |  |  |
| Transcriptomic data 1 | 51133936 | 44622044 |
| Transcriptomic data 2 | 54941426 | 48720684 |
| Transcriptomic data 3 | 48995902 | 43478296 |
| Total | 155071264 | 136821024 |
|  |  |  |
| Mycelia (PE reads, n=3) |  |  |
| Transcriptomic data 1 | 57178756 | 48903724 |
| Transcriptomic data 2 | 46200586 | 38937708 |
| Transcriptomic data 3 | 60336914 | 53139664 |
| Total | 163716256 | 140981096 |

Table S2

GO terms that are significantly expressed in fruiting bodies (Score≦5, FDR<0.05).

| GO term | Score (bit) | LogFC | FDR |
| --- | --- | --- | --- |
| RNA-dependent RNA polymerase activity | 36 | 3.74 | 0 |
| RNA processing | 34 | 1.85 | 4.2E-02 |
| sporulation | 11 | 3.24 | 1.6E-02 |
| mRNA methyltransferase activity | 5 | 8.17 | 2.1E-10 |
| mRNA methylation | 5 | 8.17 | 2.1E-10 |

Table S3

Proteins, primers, and product sizes for real-time RT-PCR.

| Protein | Forward primer | Reverse primer | Product size (bp) |
| --- | --- | --- | --- |
| Actin | CGTTACACTGTGCTCCCCTTC | CGCCGGCAAGGTTAAAGAG | 121 |
| *Pleurocybella porrigens* lectin | GTACCTCCAACAGGAGTGTTGG | GGTTACAGGCCACCCCAATAAC | 117 |
| Negative regulator of sexual conjugation and meiosis | GCTTTCAAGCAGAGCCTTGTC | CCTTTTTTCTCTCGGAGGACG | 128 |
| Polyporopepsin | CCACTTCTATCACGTTTGTCCC | GTAGTGCCAGTATCGACAATGC | 133 |
| Glucan 1,3-beta-glucosidase | CTTGCGGGTTGTTGTAGTCCTG | CTTTTTTGATGCAGGCACATACG | 131 |
| Laccase-2 | CACATTGGCTGACTGGTACCAC | GTGACAGTAATGACAGATAAAGGCG | 132 |
| Galactokinase | GTGAGCTCATCCAGCTCAGG | CTTGGTGCTGATTCCAAGGG | 119 |
| Enoyl-CoA hydratase, mitochondrial | GGATAACGATGTTGGTGCAATTG | GGTCAGATTTTGCAGGAAGTTG | 130 |
| Aldo-keto reductase | CAATGTAGGACTCTCAGAGGCC | GGTCCACCGTCAGAATTTTTC | 135 |
| Manganese peroxidase | CGTCTTTCCGGATGTGGAAC | CCAGCAAATTGGATTAAGTCGC | 132 |
| Uncharacterized amino-acid permease | GGACAACGATGATGAAAGACC | GCTTCATTTGCCACGGTCTC | 130 |
| Adenylate-forming reductase | GAATGAGCCATACTGTCCCAGAAAC | CGCCTTCTTCAATGTGTTCTG | 131 |


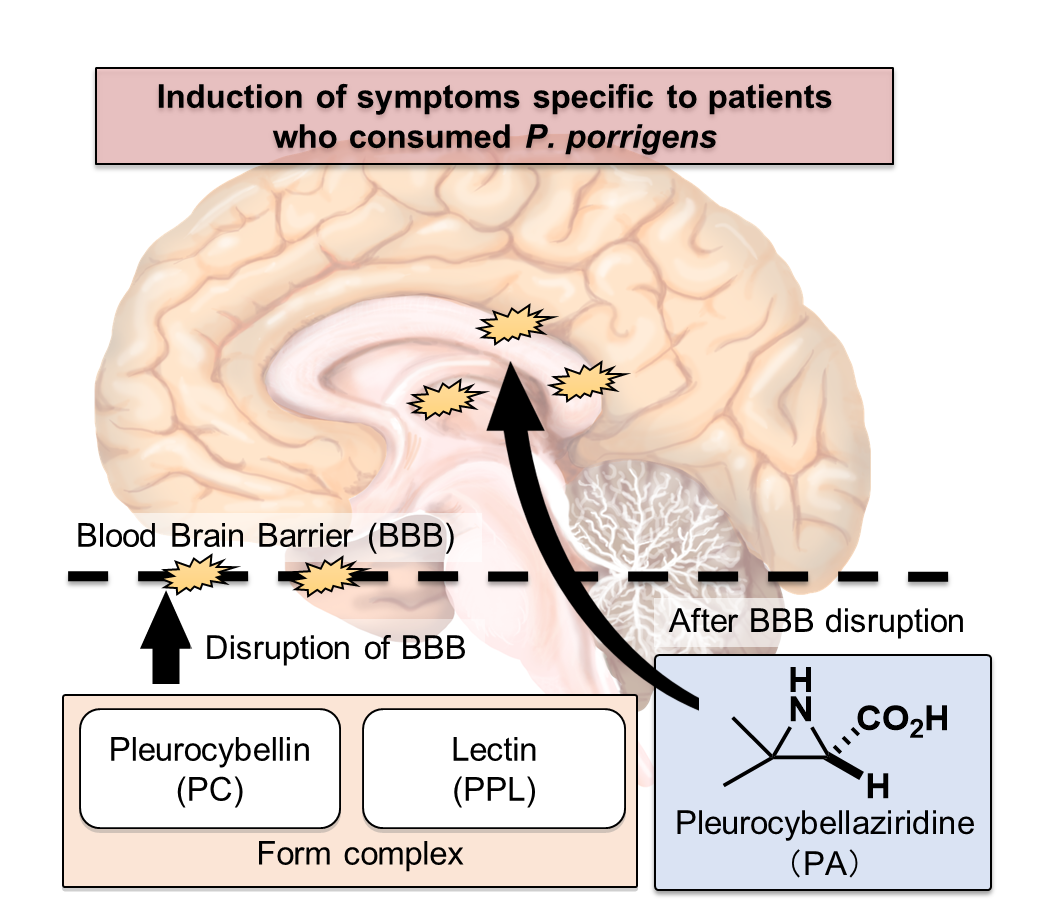


Figure S1

Proposed molecular mechanism of acute encephalopathy following ingestion of *Pleurocybella porrigens*.


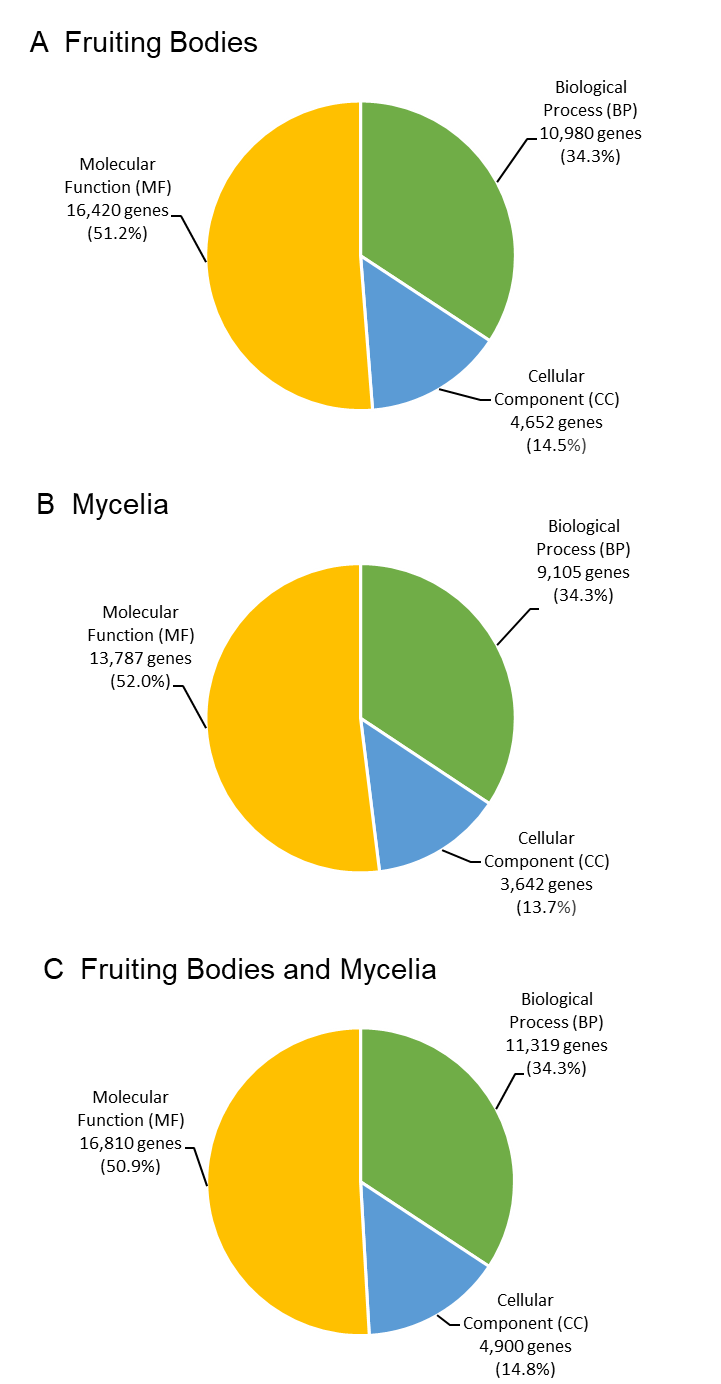


Figure S2

Distributions of GO categories assigned to the unigenes.


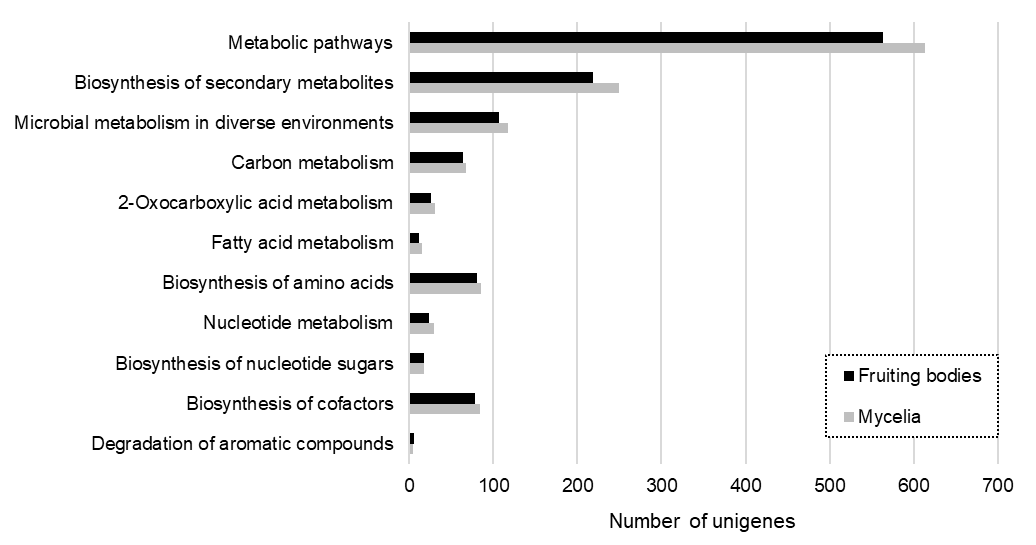


Figure S3

Distributions of KEGG assigned to the unigenes.


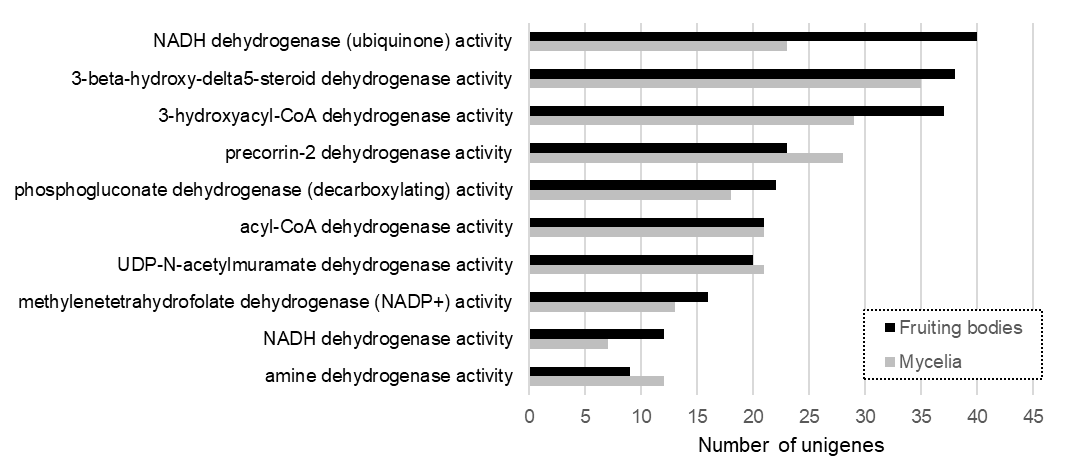


Figure S4

Dehydrogenase-related unigenes assigned to GO term (Score).

Material S1

Materials and methods: Sample preparation for RNA-seq.

Approximately 700 mg of the fruiting bodies and the mycelia of *P. porrigens* (n=3) were weighed and crushed in a mortar with liquid nitrogen. 2 mL of TRIzol (Thermo Fisher Scientific) was added and incubated at 30°C for 5 minutes. Then, 200 μL of chloroform was added, vortexed for 15 seconds, incubated at 30°C for 2 minutes, and centrifuged (12,000 × g, 15 min, 4°C). After centrifugation, 500 μL of isopropanol was added to the supernatant (water layer), incubated at 30°C for 10 minutes, and centrifuged (12,000 × g, 10 min, 4°C). The supernatant was removed to eliminate foreign substances. 1 mL of 75% ethanol was added and centrifugated (7,500 × g, 5 min, 4°C). This procedure was repeated twice, and the supernatant was removed and air-dried for 10 minutes. To the obtained total RNA pellet, 50 μL of TE buffer was added and incubated at 60°C for 10 minutes to dissolve the pellet. After measuring the concentration, 1 μL of RNase Inhibitor, Murine, was added to inhibit the remaining RNase. The total RNA was purified again using the RNeasy Plant Mini Kit. Each purified RNA was subjected to OD measurement by BioDrop, confirmation of RNA degradation by bioanalyzer (RNA 6000 nano kit), measurement of RNA concentration by Quantus, and confirmation of genomic contamination by 1% agarose electrophoresis.
